# Hemoglobin–PEG Interactions Probed by Small-Angle X-ray Scattering: Insights for Crystallization and Diagnostics Applications

**DOI:** 10.1021/acs.jpcb.4c03003

**Published:** 2024-09-10

**Authors:** Iuliia Baranova, Angelina Angelova, Jan Stransky, Jakob Andreasson, Borislav Angelov

**Affiliations:** † Extreme Light Infrastructure ERIC, Za Radnicí 835, Dolní Břežany 252 41, Czech Republic; ‡ Faculty of Mathematics and Physics, Charles University, Ke Karlovu 3, Prague 121 16, Czech Republic; § Université Paris-Saclay, CNRS, Institut Galien Paris-Saclay, F-91400 Orsay, France; ∥ Institute of Biotechnology of the Czech Academy of Sciences, v.v.i., Prumyslová 595, Vestec 252 50, Czech Republic

## Abstract

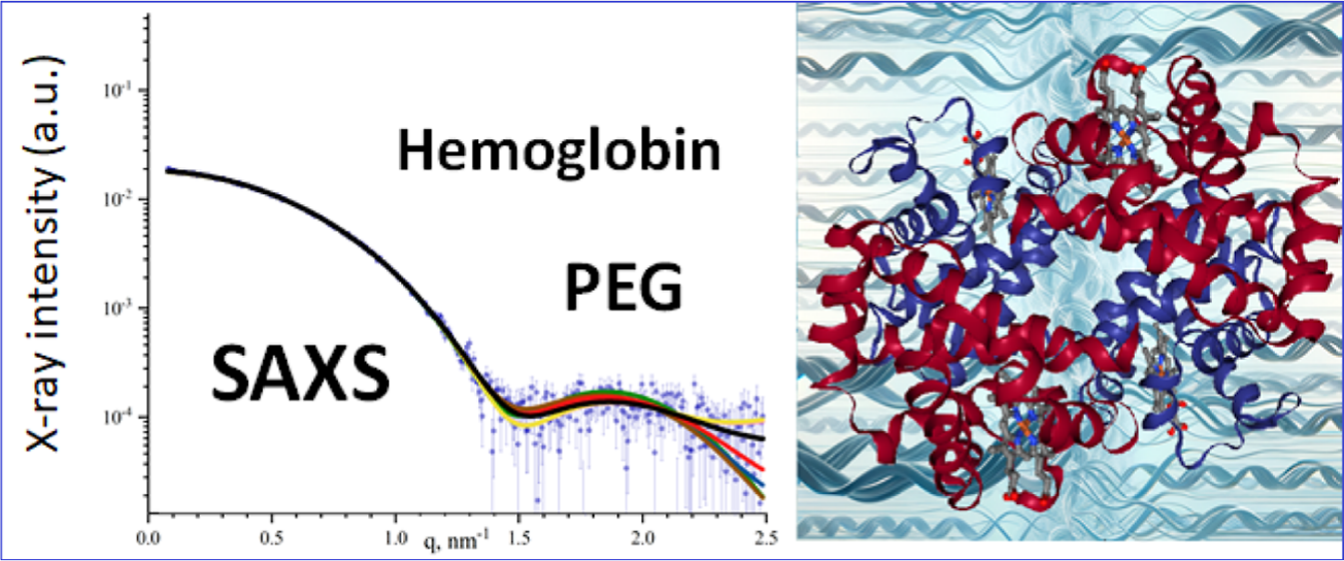

Protein–protein interactions, controlling protein aggregation in the solution phase, are crucial for the formulation of protein therapeutics and the use of proteins in diagnostic applications. Additives in the solution phase are factors that may enhance the protein’s conformational stability or induce crystallization. Protein–PEG interactions do not always stabilize the native protein structure. Structural information is needed to validate excipients for protein stabilization in the development of protein therapeutics or use proteins in diagnostic assays. The present study investigates the impact of polyethylene glycol (PEG) molecular weight and concentration on the spatial structure of human hemoglobin (Hb) at neutral pH. Small-angle X-ray scattering (SAXS) in combination with size-exclusion chromatography is employed to characterize the Hb structure in solution without and with the addition of PEG. Our results evidence that human hemoglobin maintains a tetrameric conformation at neutral pH. The dummy atom model, reconstructed from the SAXS data, aligns closely with the known crystallographic structure of *met*hemoglobin (*met*Hb) from the Protein Data Bank. We established that the addition of short-chain PEG600, at concentrations of up to 10% (w/v), acts as a stabilizer for hemoglobin, preserving its spatial structure without significant alterations. By contrast, 5% (w/v) PEG with higher molecular weights of 2000 and 4000 leads to a slight reduction in the maximum particle dimension (*D*_max_), while the radius of gyration (*R*_g_) remains essentially unchanged. This implies a reduced hydration shell around the protein due to the dehydrating effect of longer PEG chains. At a concentration of 10% (w/v), PEG2000 interacts with Hb to form a complex that does not distort the protein’s spatial configuration. The obtained results provide a deeper understanding of PEG’s influence on the Hb structure in solution and broader knowledge regarding protein–PEG interactions.

## Introduction

Polyethylene glycol (PEG) is one of the most well-known polymers that are used for various purposes in diagnostics, drug delivery, biomedical engineering, macromolecular crystallography, agriculture, and medicine. PEG is a linear, water-soluble amphiphilic polymer composed of oxyethylene monomers and can reach a molecular weight (*M*_w_) of up to several million Da. It is usually denoted as PEO for *M*_w_ above 20,000 Da. In the context of soluble proteins, PEGs have been used as biocompatible moieties for conjugation with the protein molecules, increasing their circulation times and reducing the immune response in the body.^1−3^ PEGs are also commonly used for protein precipitation and have found widespread use in protein crystallography. Analyzing Protein Data Bank (PDB) crystallographic data, Hašek has identified the major types of protein–PEG interactions: (i) creating H-bonds with the side chains of amino acid residues and the NH groups of the main protein chain, (ii) coordination of positively charged amino acid residues, and (iii) hydrophobic interactions with the proteins through the coordination of an external cation.^4^ Spectroscopic studies have shown that these interactions can affect the conformation, thermal stability, and kinetic reactions of the proteins. It is considered that proteins exclude PEGs from their hydration shells.^4,5^ This may act as a driving force for protein conformational changes.

Early works on modulating the solubility of various types of proteins by PEGs have shown that, in addition to the volume exclusion effect, there is an attractive interaction between PEG and some proteins that cannot be explained by PEG exclusion alone.^5−8^ The influence of PEGs differs in the thermal denaturation properties of different proteins. In some cases, 10% PEG 200, 400, and 1000 decrease the transition temperature of the protein, e.g., β-lactoglobulin.^5^ A decrease in transition temperature has also been observed in papain thermal denaturation experiments, where the maximum destabilizing effect has been achieved with PEG400 compared to that achieved with higher-molecular-weight PEG.^9^ The opposite, stabilizing effect of PEG has been demonstrated for α-chymotrypsin in 60% ethanol and was found to increase with increasing PEG size or PEG concentration.^10^ Under varying pH conditions, bovine serum albumin (BSA) has exhibited different behavior in the presence of PEGs. At pH 4.76 (close to pI of BSA), thermal denaturation experiments have shown that PEG400 and PEG1450 reduce the transition temperature, while PEG8000 has no effect.^11^

Intrinsic fluorescence experiments have demonstrated that there is no effect of PEG400 on BSA at various protein/polymer ratios at physiological pH. PEG4700 and PEG20000 significantly changed the Trp fluorescence, especially PEG4700, indicating an interaction between the BSA protein and polymer molecules. This has also been proven by ANS fluorescence experiments.^12^

Lyophilization and sonication experiments of BSA with PEG8000 have shown that PEG8000 stabilizes the conformation of BSA at a certain molar ratio. PEG8000 binds to BSA through a hydrophobic interaction, slightly unfolding the protein molecule. However, despite this, PEG8000 in a molar ratio of 1:0.75 (BSA/PEG8000) stabilizes BSA, which has also been proven by differential scanning calorimetry (DSC) experiments.^13^ A comparison of BSA with the related protein human serum albumin (HSA), which is slightly more hydrophilic, has shown that the interaction of PEG with BSA is dominated by hydrophobic contacts, while in the case of HSA, electrostatic interactions prevail with less disruption.^14^ Studies of myoglobin (Mb) with PEG have shown that there are interactions between PEG and Mb that lead to the formation of molten globule and premolten globule (PMG) of Mb, i.e., to destabilization of the tertiary and secondary structure of the protein.^15−18^ This destabilization depends on the molecular weight of PEG and its concentration. On the other hand, PEG has demonstrated the ability to retain heme in Mb under certain denaturation conditions.^15^ Studies of another heme protein cytochrome *c* (Cyt *c*) with PEG show that PEG forms a complex with Cyt *c* through a soft interaction on the surface of the protein.^18−21^ Moreover, PEGs with different *M*_w_s lead to slightly different changes in Cyt *c*, in some cases partially perturbing only the tertiary structure of Cyt *c*(20) and in other cases changing both the secondary and tertiary structures.^18^ In addition, PEG promotes the oxidation of Cyt *c* by molecular oxygen.^22^ It has been suggested that heme oxidation is caused by small changes in the tertiary structure and a small shift in the position of the heme, which is negligible in the absence of PEG.

Hemoglobin is the protein responsible for transporting oxygen into the blood. Studying the solution structure of hemoglobin and its interactions with poly(ethylene glycol) are important for several reasons. First, understanding the Hb structure and how it behaves in solution is crucial for medical applications, particularly in blood transfusion and the development of blood substitutes. It should be noted that hemoglobinopathies comprise disorders affecting the hemoglobin structure or function (e.g., sickle cell disease). Second, hemoglobins are among the most extensively studied groups of heme proteins. They have demonstrated the ability to crystallize using PEG^23−31^ and are of interest for studying PEG–protein interactions. Studying the interaction between Hb and PEG can lead to more stable hemoglobin-based therapeutics with increased shelf life and reduced immunogenicity.

In the present study, we investigate the influence of PEGs on human Hb at different concentrations of PEG (5, 10, and 20% w/v) and different molecular weights of PEG (600, 2000, and 4000) by biological solution small-angle X-ray scattering (BioSAXS). Understanding the structural changes in Hb in response to PEG surroundings has implications for the treatment and diagnosis of diseases where the hemoglobin structure is altered or where hemoglobin is used as a biomarker. The study of Hb–PEG interactions can also contribute to our understanding of protein chemistry and biophysics such as how macromolecules behave in different environments and how modifications can affect protein structure and function.

## Materials and Methods

### Hemoglobin Sample Preparation

Lyophilized human hemoglobin (Sigma-Aldrich) was dissolved in a 100 mM sodium phosphate (NaP) buffer. This buffer was prepared by mixing disodium hydrogen phosphate (Na_2_HPO_4_) and sodium dihydrogen phosphate (NaH_2_PO_4_) from Sigma-Aldrich in a specified ratio. The pH of the solution was then fine-tuned to 7.0 using 4 M hydrochloric acid (HCl) from Sigma-Aldrich and 1 M sodium hydroxide (NaOH) from PENTA (Prague, Czech Republic).

The Hb solution was centrifuged twice, each time for 20 min at 18,000 times gravity (*g*) at a temperature of 4 °C. Subsequently, the solution was filtered through a 0.22 μm polyvinylidene fluoride (PVDF) membrane filter. From this solution, 500 μL of sample with a concentration of roughly 18 mg/mL was loaded into a GE Superdex 75 Increase 10/300 chromatographic column (Sigma-Aldrich) connected to an AKTA Go liquid chromatography system by GE Healthcare (Germany) for size-exclusion chromatography (SEC) coupled with small-angle X-ray scattering (SEC-SAXS) analysis.

After the initial SEC-SAXS characterization, the separation process was repeated thrice, and fractions corresponding to the same elution peak were combined. This pooled sample was then concentrated to a final concentration of 28.4 mg/mL, as determined by a multicomponent spectrophotometric method.^32^ According to the UV–vis spectrum, about 86% of Hb was in the oxidized state (*met*Hb) (Figure S2).

PEGs of various molecular weights PEG600, PEG2000, and PEG4000 (Sigma-Aldrich) were dissolved in the same 100 mM NaP buffer, and the pH was adjusted to 7.0 using 4 M HCl and 1 M NaOH. For the SAXS measurements, the samples consisted of 5 mg/mL Hb in the 100 mM buffer, with PEG concentrations at 5, 10, and 20% (w/v).

For synchrotron SAXS experiments, samples were prepared by dissolving Hb in a 100 mM NaP buffer. The pH of this buffer was adjusted from 6.8 to 6.9. The Hb solution was centrifuged as described above to remove any insoluble material, but unlike previous preparations, this solution did not undergo SEC. PEGs with different molecular weights—PEG600, PEG2000, and PEG4000—were also prepared in a 100 mM NaP buffer with a pH adjusted to the same range from 6.8 to 6.9 as the Hb solution. The concentration of PEG in the samples was standardized to 5% by weight/volume (w/v), and the pH was fine-tuned to 6.9. The final samples contained Hb at a concentration of 5 mg/mL, ensuring consistency across all synchrotron SAXS experiments.

### SAXS and SEC-SAXS on a Laboratory Instrument

Laboratory SAXS experiments were carried out using a SAXSpoint 2.0 system from Anton Paar, Austria. This instrument features a high-intensity MetalJet C2+ X-ray source from Excillum, Sweden, which produces X-rays with a wavelength of 1.34 Å. The size of the X-ray beam incident on the sample was specified as 982.7 μm^2^, and the beam had an intensity, or flux, of approximately 10^8^ photons per second. X-ray images were acquired with an EIGER 1 M detector from Dectris, Switzerland. The distance between the sample and the detector was 792.6 mm in SEC-SAXS measurements and 825.6 mm in the usual SAXS measurements. The system was also equipped with an automatic sample delivery mechanism, which included a UV–vis stage, Cary UV 60 from Agilent, and a liquid chromatography system, the AKTA Go from GE Healthcare. The acquisition time for each SAXS frame was 15 s, and during this time, UV–vis spectra were recorded simultaneously to monitor the sample.

The data from the experiments were processed by using several software tools. CHROMIXS,^33^ the PRIMUS program, and the DAMMIF/N program, which are parts of the ATSAS 3.2.0 software suite,^34^ were all employed for the analysis. Data from the SAXS and SEC-SAXS experiments were normalized to the primary beam intensity using a semitransparent beamstop. SAXS data from the SEC-SAXS experiment were subtracted and fitted against various crystallographic models from the PDB using the CRYSOL program (ATSAS 3.3.0). Twenty spherical harmonics, 501 points, 0.361 e/Å^–3^ solvent density (corresponding to 100 mM NaP buffer pH = 7.0) with default contrast of the solvation shell 0.03 e/Å^–3^, directional shell kind, and 18 Fibonacci grid were applied for every calculation. In addition, SAXS data were compared and superimposed onto a known crystallographic structure using the SUPCOMB tool, which is integrated into the BioXTAS RAW software.^35^ For visualizing the three-dimensional models obtained from these analyses, the Chimera program^36^ was used. This software allows for detailed viewing and manipulation of complex molecular structures and is a standard tool in the field for such purposes.

The measurements of Hb samples, both with and without the addition of PEG, were conducted by utilizing a 1 mm quartz capillary. To ensure consistency during the experiments, the samples were thermally stabilized at a temperature of 293 K (i.e., approximately 20 °C). For every sample, a total of 30 frames were collected. To refine the raw data, we subtracted the contribution of the buffer solution from the sample scattering using the PRIMUS program.^34^

Furthermore, we performed a Guinier analysis to determine the radius of gyration (*R*_g_), which is a measure of the particle size. The maximum particle dimension (*D*_max_) from the pair-distance distribution function *P*(*r*) was calculated with the PRIMUS program. Kratky plots, which are graphical representations used to study the structural conformation and compactness of biological macromolecules in solution, were generated using the BioXTAS RAW software. In order to create 3D reconstructions of the Hb molecules, we employed the DAMMIF program in slow mode with 20 reconstructions that then were averaged with DAMAVER and refined with DAMMIN from the ATSAS 3.2.0 package. These reconstructions are based on dummy atom models, which enable simplified representations of the biomacromolecules with information about their overall shape and structure.

### Synchrotron SAXS

Hb solution samples were analyzed at the ID02 beamline of the European Synchrotron Radiation Facility (ESRF) (Grenoble, France). The samples were measured using flow-through capillaries of 1.5 mm in diameter. The X-ray beam size on the samples was 50 × 120 μm (vertical and horizontal dimensions, respectively). The X-ray beam wavelength was set to 1 Ångström (Å), and the beam flux was around 10^12^ photons per second on the sample. The data were collected with an acquisition time of 0.1 s for every frame. For the average, 20 frames were acquired along the flow-through capillary length. After the data collection, the scattering curves of the samples were processed to remove the background contribution of the buffer solution (PRIMUS program).

All figures in the article were plotted using OriginPro (version 2020, OriginLab Corporation, Northampton, MA, USA).

### Molecular Dynamics Simulations

The molecular dynamics (MD) simulations were conducted using the WAXSiS online server^37^ at University of Saarland, Germany (https://waxsis.uni-saarland.de). This online server needs an initial PDB file (PDB ID: *3odq.pdb* in our case) to compute the desired SAXS/WAXS curves of hemoglobin in solution. The calculations are based on all-atom MD simulations that explicitly model the solvent environment. The solvation shell around hemoglobin is presented in atomic detail, capturing the thermal fluctuations and dynamic nature of the hydration layer. In contrast to methods based on the implicit solvent (dummy atoms as in DAMMIN), WAXSiS does not require any solvent-related fitting parameters such as the density of the hydration layer, overall excluded solvent, or scaling parameters for dummy atoms. WAXSSiS uses an AMBER03 force field implementation for the protein^37^ and the TIP3P water model for the water solvent.^37^ The total duration of the MD simulation was 55 ps, with the initial 3 ps dedicated to the equilibration phase. The simulation system contained 9080 solute atoms and 17,033 water molecules. The further step performed by the WAXSiS server was to fit the experimental SAXS curve to the SAXS/WAXS curve calculated from MD simulations. This fitting procedure uses two adjustable parameters: (a) the overall scale, which is arbitrary in nature, and (b) a constant offset that helps absorb experimental uncertainties arising from buffer subtractions. These parameters allow the calculated curve to be adjusted for the best fit with the experimental data while accounting for scaling differences and baseline shifts that can occur in SAXS experiments.^37^ Supporting Information Figures S4 and S5 show the fit of the experimental SAXS data to the MD calculated SAXS/WAXS curves and the visualization of the hemoglobin in water solution atomic models.

## Results

### SEC-SAXS Characterization of Human Hemoglobin

Hb under the studied solution conditions was characterized using a combination of SAXS and SEC. SEC-SAXS is a powerful new technique used in the analysis of biomacromolecules. While SAXS provides information about the size, shape, and orientation of protein particles, SEC, also known as gel filtration, is a type of chromatography that separates molecules based on their size (or more accurately, their hydrodynamic volume). During the SEC run, the larger molecules elute first, while smaller molecules take longer to come out of the column. When SAXS is combined with SEC, a sample is first separated into its constituent parts by SEC. The separated fractions are then analyzed by SAXS. This combination allows one to obtain detailed information about the shape and size of individual components within a complex mixture and is particularly valuable in studying Hb proteins and their complexes in solution. The combined SEC-SAXS method has advantages, such as the reduction of sample polydispersity, the removal of aggregates, and the reduction of the probable X-ray degradation effect due to refreshing the sample during the measurement. This technique can provide valuable insights into the structural changes and interactions of Hb with PEG as well.

**Figure 1:**
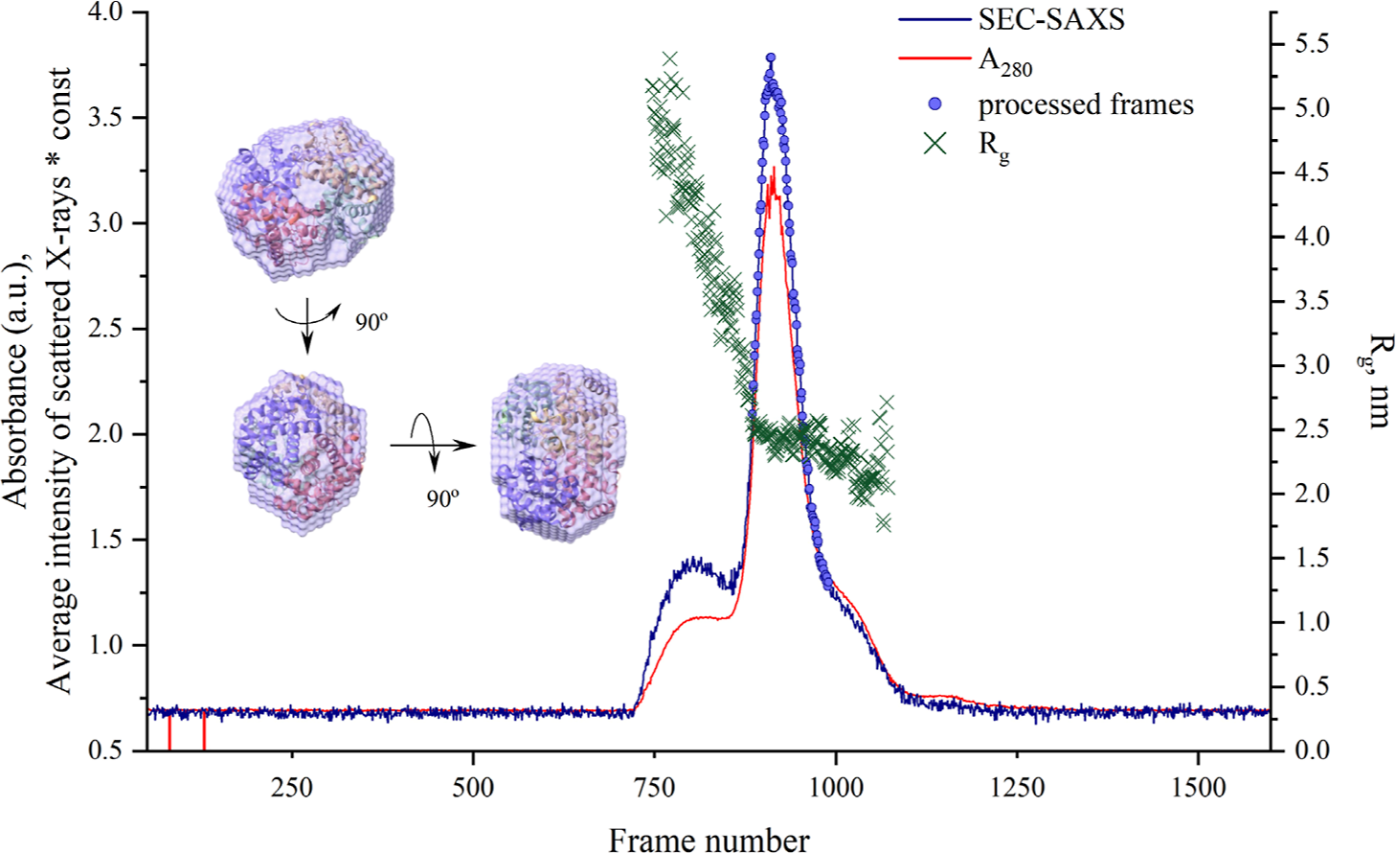
SEC-SAXS analysis of a human hemoglobin sample in 100 mM NaP buffer at pH 7.0. The dark blue solid line represents integrated intensities (in arbitrary units) multiplied by a constant factor of 15, combined with UV absorbance (in arbitrary units) at 280 nm (red solid line) plotted against frame number. The radii of gyration frames of two peaks, estimated by the CHROMIXS program, are indicated by dark-green crosses. Selected frames for further processing are depicted as filled purple circles. A reconstructed dummy atom model derived from the selected SEC-SAXS data was generated using the DAMMIF/N program and superimposed on the crystallographic structure of *met*hemoglobin (*3odq*) using the SUPCOMB program. The resulting models are visualized in the Chimera program, and the overlay of the two models is presented in three different orientations.

**Figure 2:**
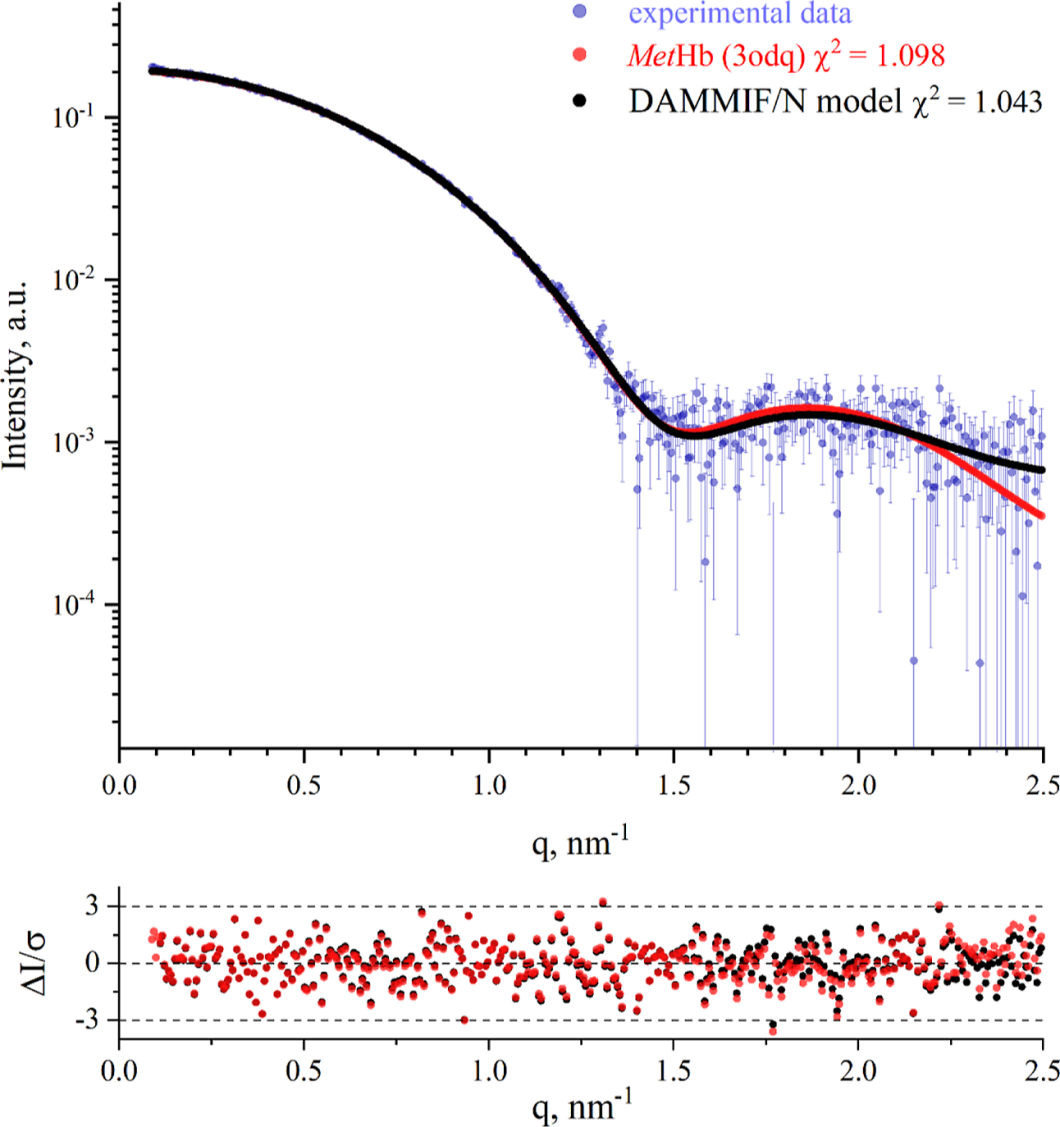
Averaged experimental SEC-SAXS data of the second elution peak are represented by light purple circles. The theoretical scattering curves of the crystallographic structure *3odq* obtained from PDB are calculated and fitted to the experimental data using CRYSOL, shown as red circles. The ab initio model generated by DAMMIF/N is presented as black circles. The upper plot shows the log-scale plot of *I*(*q*) versus *q*, while the lower inset plot illustrates the error-weighted difference between the model and experimental data.

Figure 1 illustrates the elution profile of the Hb sample components over time, where each frame corresponds to an exposure time of 15 s. The solid red line depicts the absorbance at 280 nm, indicative of protein presence, while the solid dark blue line shows the averaged SAXS intensity across a *q*-range of 0.1–0.8 (1/Å) for each time point. The absorption profile is characterized by two main peaks and a shoulder on the larger peak. In a similar way, the SAXS data have two main peaks and a shoulder corresponding to different protein fractions. The radius of gyration (*R*_g_) for the two peaks and the shoulder, represented by dark green crosses in Figure 1, was calculated using the CHROMIXS program. The first elution peak exhibited a high dispersion in the radius of gyration (*R*_g_) values. Therefore, further analysis was focused on the second elution peak, which had a smaller dispersion of the *R*_g_. Subsequently, the buffer was subtracted, and the resulting data were fitted against various crystallographic models from the PDB using the CRYSOL program. These models included *2dn1* (human *oxy*Hb),^38^*2dn2* (human *deoxy*Hb),^38^*3odq* (human *met*Hb indicative of fiber formation),^25^*3p5q* (human R-state *aquomet*Hb),^39^*6nbc* (human *met*Hb state1 determined using single-particle cryo-EM),^40^ and *6nbd* (human *met*Hb state 2 determined using single-particle cryo-EM)^40^ (Figure S1). The *3odq*, corresponding to a fiber-forming state of Hb, showed the closest fit to the experimental data, with a chi-squared (χ^2^) value of 1.098. It is presented in Figure 2 along with the simulated scattering curve from the ab initio model, reconstructed using the DAMMIF/N program. The alignment of the *3odq* PDB model with the SAXS-derived ab initio model is illustrated in Figure 1 in three different orientations, achieved using SUBCOMB and visualized with the Chimera program.

The SAXS analysis indicates that the predominant form of Hb in the sample, prepared in sodium phosphate (NaP) buffer at pH 7.0, is tetrameric with a *R*_g_ of 2.38 nm and a maximum diameter (*D*_max_) of 6.8 nm. Although there are higher-order oligomers present, their quantity is minor. However, the size dispersion of the first elution peak is notable, which complicates the analysis with the current design of the experiment. The UV–vis absorption spectrum of the sample confirms that Hb is primarily in the oxidized *met* hemoglobin (*met*) state (see Figure S2).

**Figure 3:**
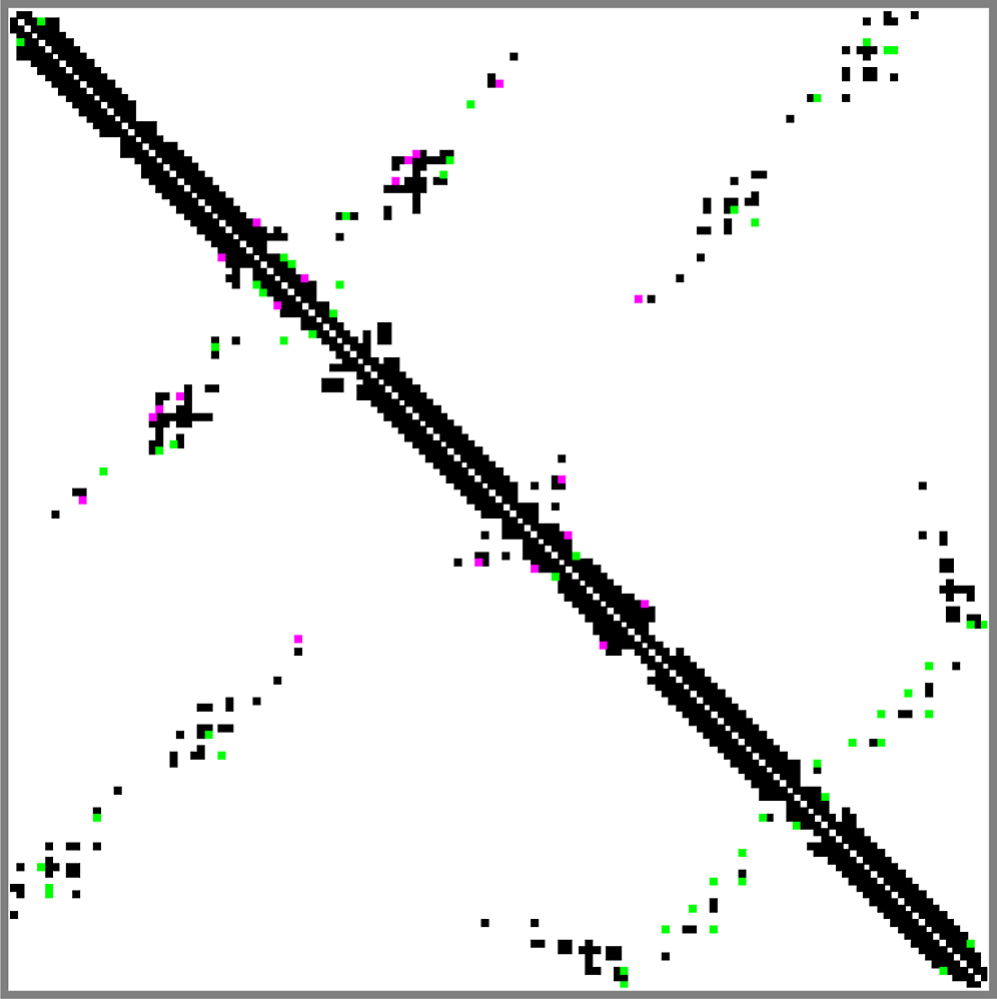
Contact maps for the solution and crystallographic structure (PDB ID*: 3odq*) of the hemoglobin. Black small squares show the contacts that belong to both structures; magenta ones are unique for the solution structure and the green ones are unique for the crystalline structure.

To enhance the precision of comparisons between hemoglobin solution and crystal structures, we analyzed the protein’s contact maps. These maps graphically represent the proximities between amino acid residues within Hb and can be derived via multiple computational approaches referenced in the literature.^41,42^ Our investigation utilized a cutoff-based method that relies on the atomic coordinates of the protein and implemented in the program CMView.^42^ The initial low-resolution model, constructed with dummy atoms, lacked accurate atomic details. To overcome this, MD simulations were employed to refine the protein structure and adjust it to the SAXS data obtained from the solution studies. The Hub group has established an online platform for conducting such detailed explicit-solvent all-atom MD simulations.^37^Figure 3 presents the obtained contact maps for a single Hb chain, demonstrating substantial congruence with a 94.4% overlap between the compared structures. In the MD solution-derived structure, 635 contacts were identified with 10 unique contacts marked by magenta squares in Figure 3. The crystal structure (PDB ID: *3odq*), in contrast, displayed 652 contacts, including 27 unique contacts denoted by green squares. There were 625 contacts common to both structures. The high number of contacts in the crystallographic structure could be indicative of more compact packing of the amino acids.

### Influence of the PEG600 Concentration on the Structure of Human Hemoglobin

PEG600 could affect the human hemoglobin structure through multiple mechanisms, including the excluded volume effect, altered protein solvation, or specific molecular interactions.^5^ The effect of PEG600 on the hemoglobin structure was investigated using SAXS.

**Figure 4:**
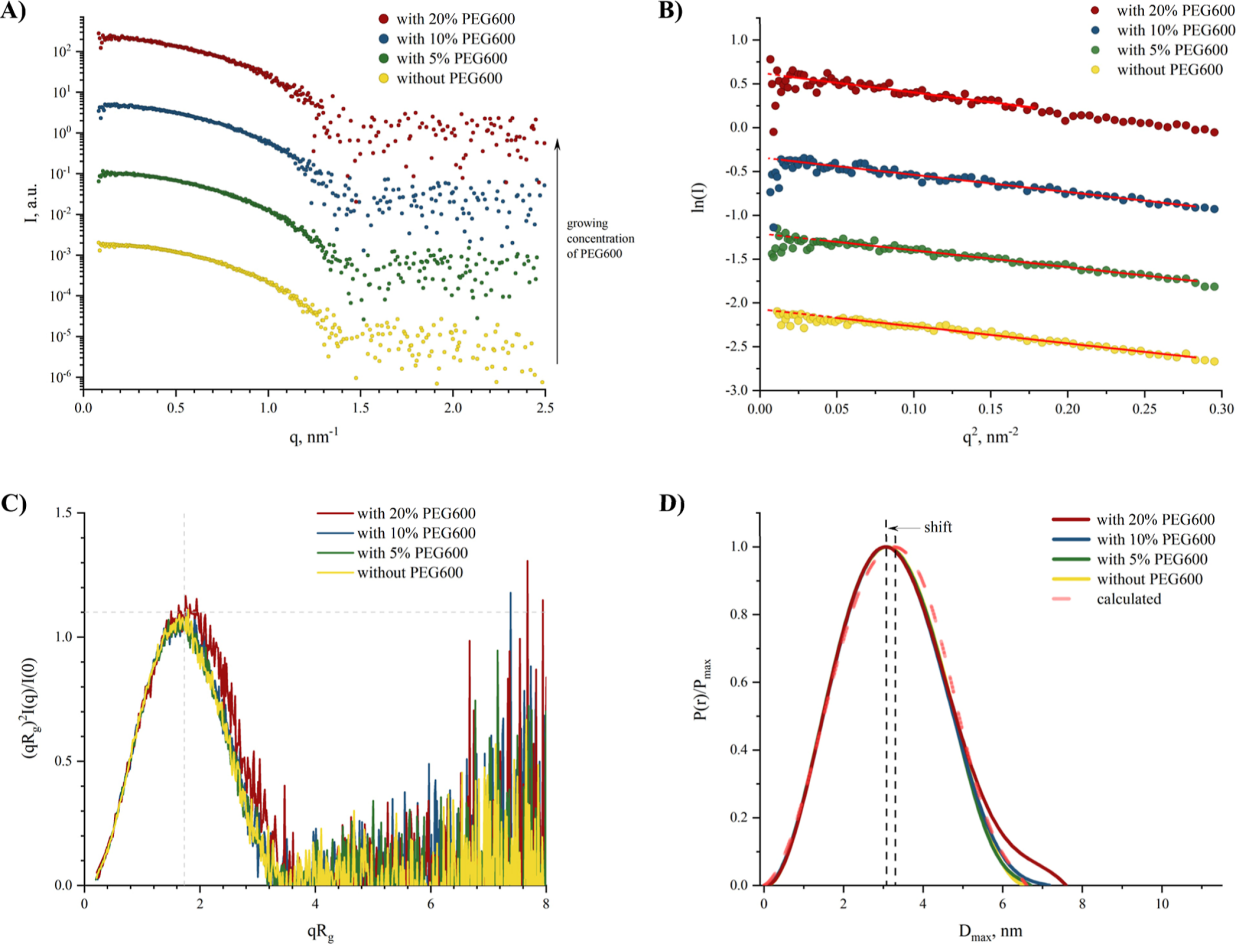
SAXS analysis of a human hemoglobin sample in 100 mM NaP buffer at pH = 7.0 with different concentrations of PEG600: (A) experimental data after subtraction of buffer with PEG600 shifted along the *I*(*q*) axis (log scale); (B) Guinier plot for the data with the linear fits (red lines); (C) dimensionless Kratky plots; (D) normalized to the maximum value *P*(*r*) versus *D*_max_; red dashed line demonstrates the *P*(*r*) function calculated for the *3odq* crystallographic structure in CRYSOL.

Figure 4A presents the SAXS data for Hb samples in the presence of PEG600, following buffer subtraction, which contained an equivalent concentration of PEG600. These samples were all prepared using Hb from the second peak of elution and in the same NaP buffer as employed for SEC-SAXS experiments. The SAXS data reveal a lack of significant curvature in the low-*q* region for each sample.

The radius of gyration *R*_g_ was determined using the Guinier approximation, and the corresponding Guinier plots are depicted in Figure 4B. These plots include the linear fits, and the derived *R*_g_ values are listed in Table 1. The concentrations of 5% (w/v) and 10% (w/v) PEG600 did not markedly influence the Hb molecule size. However, increasing the PEG600 concentration to 20% (w/v) resulted in an *R*_g_ alteration of 1.7 Å, which exceeds the experimental error margin.

**Table 1:** Parameters Derived from SAXS Measurement of Hb without and with PEGs in 100 mM NaP Buffer at pH = 7.0

sample	*I*(0) from Guinier, a.u	*R*_g_ from Guinier, nm	*I*(0) from *P*(*r*), a.u	*R*_g_ from *P*(*r*), nm	*D*_max_, nm
Hb after 1 day	0.1300 ± 0.0009	2.41 ± 0.02	0.1260 ± 0.0006	2.397 ± 0.009	6.65
Hb with 5% (w/v) PEG600	0.1100 ± 0.0007	2.39 ± 0.02	0.1103 ± 0.0005	2.399 ± 0.008	6.71
Hb with 10% (w/v) PEG600	0.0960 ± 0.0008	2.43 ± 0.03	0.0960 ± 0.0005	2.417 ± 0.011	7.18
Hb with 20% (w/v) PEG600	0.0690 ± 0.001	2.57 ± 0.08	0.0671 ± 0.0004	2.524 ± 0.014	7.6
Hb after 1 week	0.1200 ± 0.0007	2.40 ± 0.02	0.1205 ± 0.0004	2.416 ± 0.007	6.8
Hb with 5% (w/v) PEG2000	0.110 ± 0.001	2.41 ± 0.03	0.1083 ± 0.0004	2.358 ± 0.007	6.5
Hb with 5% (w/v) PEG4000	0.0970 ± 0.0007	2.39 ± 0.03	0.0960 ± 0.0004	2.318 ± 0.008	6.12
Hb with 10% (w/v) PEG2000	0.0820 ± 0.0008	2.83 ± 0.04	0.0828 ± 0.0008	2.912 ± 0.043	11.3

Dimensionless Kratky plots, illustrated in Figure 4C, show bell-shaped curves for all Hb samples, indicating a globally compact state. The maximum near a *qR*_g_ value of √3 is consistent with a theoretical model of a compact globular protein. The peak for the Hb sample with 20% (w/v) PEG600 aligns well with this theoretical value, whereas for Hb samples without PEG600 or those with 10% (w/v) PEG600, the curves are slightly lower and the peak positions modestly shifted to lesser values, suggesting a less symmetric shape for these Hb samples compared to the one with 20% (w/v) PEG600. The pair distance distribution functions, *P*(*r*), for each sample are shown in Figure 4C. All functions display a maximum within the range of *D*_max_ ≈ 3.02–3.09 nm, deviating from the theoretical *D*_max_ ≈ 3.25 nm derived from the crystal structure (PDB ID: *3odq*), which is indicated by a transparent red line in Figure 4C. The *P*(*r*) for Hb in the presence of 20% (w/v) PEG600 declines differently from the others, a discrepancy likely due to the specific arrangement of PEG600 molecules around the Hb molecule. This arrangement is not present in the buffer containing 20% (w/v) PEG600 alone. The *D*_max_ used during data processing was chosen as the point beyond which the *P*(*r*) function plateaued, and values beyond this point were set to zero. The parameters extracted from the *P*(*r*) functions are compiled in Table 1 and demonstrate good agreement with the values obtained from the Guinier analysis. The dependency of *R*_g_ on the concentration of PEG600 is further explored in Figure S3.

PEG600 is known to play a significant role in the stabilization of specific protein conformations. In the context of Hb, it can induce conformational changes through a combination of direct interactions with the protein and indirect mechanisms such as exclusion volume effects. Additionally, the incorporation of PEG600 into the solution can modify the solvent characteristics, which in turn could impact the hydrogen bonding patterns and hydrophobic interactions within the Hb molecule. These alterations in solvent properties have the potential to exert an influence on the tertiary and quaternary structural arrangements of the protein, thereby affecting its stability and function. The temperature, pH, and ionic strength should also be taken into account when considering the PEG600 concentration effects.

### Influence of the PEG Size on the Structure of Hemoglobin

The influence of PEG molecular weight, while keeping the concentration fixed, on the structure of Hb involves a number of considerations related to the interactions between PEG molecules and the protein. The potential effects of varying the PEG size on the Hb structure and function depend on the intricate balance between the different forces at play, such as hydrophobic interactions, hydrogen bonding, electrostatics, and steric effects.

**Figure 5:**
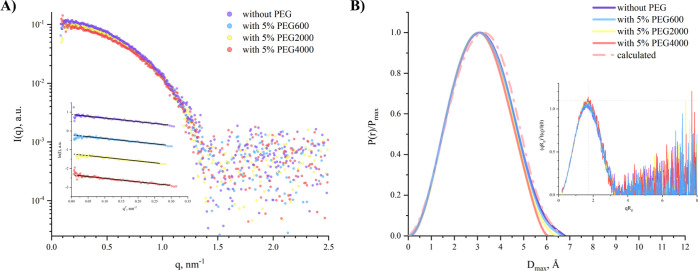
SAXS analysis of a human hemoglobin sample in 100 mM NaP buffer at pH = 7.0 with 5% (w/v) PEG600/2000/4000: (A) experimental data after subtraction of buffer with PEGs [*I*(*q*) in log scale versus *q*] and inset showing the Guinier fits (black solid line); (B) normalized to the maximum value *P*(*r*) versus *D*_max_ and inset showing dimensionless Kratky plots.

To investigate the influence of PEG molecular weight on the spatial structure of Hb, SAXS data were compared for Hb in the presence of 5% (w/v) PEG with molecular weights (*M*_w_) of 600, 2000, and 4000, as shown in Figure 5. The Guinier plots confirm the absence of aggregates in the samples and indicate that the radius of gyration (*R*_g_) of Hb slightly decreases with the increase in PEG size, as summarized in Table 1. This trend is more pronounced when *R*_g_ values are derived from *P*(*r*). The difference between the *R*_g_ values obtained from the Guinier approximation and those from *P*(*r*) increases with the PEG molecular weight, although this could be attributed to noise in the data.

Dimensionless Kratky plots, provided in Figure 5B (inset), exhibit bell-shaped curves that align closely with the theoretical values expected for a spherical compact molecule. The *P*(*r*) functions, characterized by a single peak, suggest monodispersity of the samples. An increment in PEG size results in a reduced *D*_max_, yet these values remain close to the *D*_max_ of 6.61 nm calculated from the crystallographic structure of Hb (PDB ID: *3odq*).

**Figure 6:**
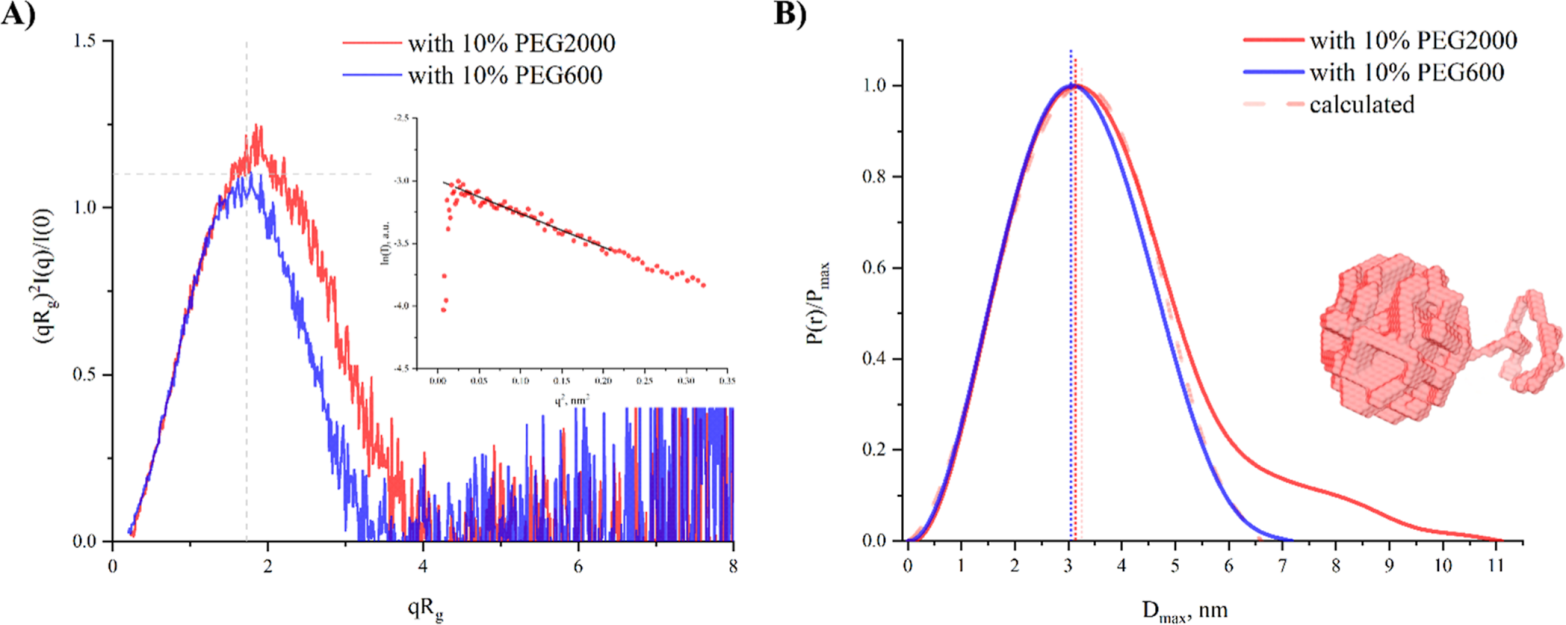
Comparing the Hb with PEG2000 and PEG600 at 10% (w/v) concentration in 100 mM NaP buffer at pH = 7.0: (A) Dimensionless Kratky plots with Guinier plot (inset). (B) Normalized to the maximum value *P*(*r*) versus *D*_max_ and the most representative 3D dummy atom model reconstructed by DAMMIF.

Figure 6 compares the effects of 10% (w/v) concentrations of PEG2000 and PEG600. The dimensionless Kratky plots show that both Hb samples with PEG2000 and PEG600 maintain bell-shaped curves, although the peak height and position for Hb with PEG2000 deviate more substantially from the theoretical model of a spherical compact molecule. The *P*(*r*) functions reveal more distinct differences; the *P*(*r*) for Hb with PEG2000 displays a shift of the main peak toward larger distances and an elongated tail with a noticeable shoulder. Considering the linear Guinier plot from scattering data at low-*q* values (Figure 6A inset) that affirms the monodispersity of the samples, this shoulder could suggest specific interactions between PEG2000 and Hb or a unique organization of the PEG polymer chains compared to that of the PEG in buffer alone. The most representative three-dimensional reconstruction of the Hb-PEG2000 complex at 10% (w/v) concentration is presented in Figure 6B.

**Figure 7:**
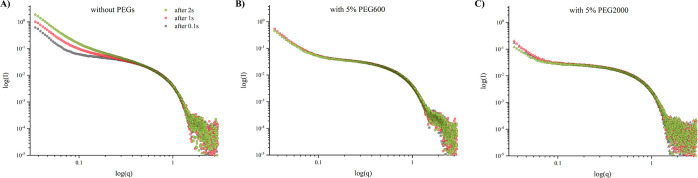
Log–log plot of SAXS data of Hb without SEC separation in 100 mM NaP buffer at pH = 6.9 with and without PEGs.

Figure 7 shows SAXS data for Hb samples with and without PEGs, obtained at the ID02 beamline of the ESRF. Notably, the Hb used in these experiments was only centrifuged and not subjected to SEC. As a result, the data exhibit differences from those obtained with SEC-SAXS. Nevertheless, a comparative analysis of the subtracted data reveals that in the absence of PEGs, the scattering curves exhibit an increase at the low-*q* range over time under X-ray exposure, while the samples containing PEGs demonstrate enhanced stability during the measurement period. This observation suggests that PEGs may also provide a protective effect against X-ray-induced aggregation of Hb.

## Discussion

### PEG Size, Concentration, and Excluded Volume Effects

Excluded volume effects significantly impact the behavior of proteins in solutions, especially when mixed with PEGs.^48^ These effects occur because two molecules cannot share the same space simultaneously. When PEG molecules are added to a solution, they occupy a considerable volume, effectively reducing the available space for protein molecules.^49^ This reduction in accessible volume increases the effective concentration of proteins, despite their molecular count remaining constant.^49^ Higher PEG concentrations amplify this effect, further confining proteins into a smaller accessible volume.^48^ Consequently, the volume available for each molecule decreases, affecting their interactions, conformational flexibility, and diffusion.^49^ This crowding effect can significantly influence protein behavior, such as solubility, stability, and aggregation or crystallization tendencies.^48^

Steric effects are a subset of excluded volume effects that specifically refer to the influence of the spatial arrangement of atoms or groups within a molecule on the molecular reactivity and interactions. Steric hindrance occurs when the size and shape of groups within a molecule prevent chemical reactions or physical interactions that would otherwise be possible. PEG can cause steric hindrance when it binds to or interacts closely with proteins like Hb. Its flexible chain can wrap around or interact with the protein surface, potentially blocking access to active or binding sites and influencing the protein structure and function. Excluded volume effects are generally considered at the scale of whole molecules and their overall volume in solution, whereas steric effects are typically at the atomic or molecular group scale.^49^

In addition to excluded volume effects, the size of the PEG influences its interaction with the protein surface by affecting conformations and the solubility. Larger PEG molecules can increase solution viscosity, slowing diffusion of protein, and may reduce the solubility, leading to protein precipitation. Furthermore, the osmotic pressure and hydration dynamics around the protein molecule are affected by PEG size, with potential impacts on the protein thermal stability and structural properties.^49^

PEGs of identical molecular weights can have completely opposite effects on various proteins. Our initial investigation focused on the influence of low-molecular-weight PEG600 on Hb at several PEG concentrations. Increasing PEG600 to a 10% (w/v) concentration did not affect the radius of gyration (*R*_g_), while the maximum particle dimension (*D*_max_) increased slightly, by approximately 0.5 nm. At a 20% (w/v) concentration, *D*_max_ extended by nearly 1 nm, and *R*_g_ exhibited a subtle increase of about 0.15 nm, small yet beyond the margin of error. This suggests that Hb shape might be altered at this concentration, as also supported by a more spherical Kratky plot compared to that of pure Hb. Nonetheless, the maximum of the pair distribution function (*P*(*r*)) for Hb with 20% (w/v) PEG600 remains similar to that of pure Hb, indicating that any changes in Hb shape due to 20% (w/v) PEG600 are not pronounced.

Previous spectroscopic studies of low-molecular-weight PEG400 effects on myoglobin (Mb, *M*_w_ ≈ 17 kDa) and BSA (*M*_w_ ≈ 66,5 kDa) at physiological pH (7.0–7.4) revealed significant alterations in Mb secondary and tertiary structures, while the BSA conformation remained largely unaffected.^12,17^ The Hb tetramer has a similar molecular weight to BSA, but the Hb monomer is comparable to Mb in structure and heme group. The four heme pockets within the Hb tetramer are less exposed than the single heme pocket in monomeric Mb, making them less accessible to short PEG chains. In our study, PEG600 impact on Hb was minimal, akin to PEG400 effect on BSA. The observed *D*_max_ increase of Hb at 20% (w/v) PEG600 could be due to changes of the water content around the protein at high concentrations of low-molecular-weight PEGs.^43^ However, this result is uncertain due to potential incomplete buffer subtraction, resulting from different scatterings of PEG with and without Hb. These findings require further investigation, possibly through small-angle neutron scattering (SANS), to discern the sample components separately.

Our secondary objective of this study was to examine the effect of PEG size on Hb. We evaluated Hb with 5% (w/v) concentrations of PEG600, PEG2000, and PEG4000. The data showed that larger PEG sizes resulted in a minor decrease in *D*_max_, with *R*_g_ values remaining within the margin of error. This subtle *D*_max_ reduction might be attributable to the varying hydrophilic properties of the PEGs. Research by Reid and Rand indicates that higher-molecular-weight PEGs bind more water per gram, suggesting that larger PEGs might isolate Hb from water molecules more effectively, potentially causing slight protein compression.^43^

Comparing samples with 10% (w/v) PEG600 and PEG2000 demonstrated more pronounced interactions between PEG molecules and Hb at higher PEG concentrations. The dummy atom model in Figure 6 represents a formed core with a chain twisted at some distance. According to the *P*(*r*) function, the size of the main core approximately coincides with the linear dimensions of the Hb molecule to some extent (if we prolong the main peak, it reaches zero between 7.5 and 8 nm). The size of the remaining part of *P*(*r*) attributed to the chain in the 3D reconstruction is about 3–3.5 nm. The Flory radius (*R*_F_), or the end-to-end distance of a polymer chain in a good solvent, is described by the equation
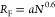
where *a* is the effective length of a polymer unit and *N* is the number of units. PEG2000, comprising approximately 45.45 oxyethylene units, each 3.5 Å long, has a *R*_F_ of 3.46 nm in a good solvent, aligning with the *P*(*r*) value obtained. The radius of gyration (*R*_g_) for PEG2000 from SANS experiments is 1.37 nm at 1% concentration without salts and 1.49 and 1.21 nm at 0.5% concentration with 10 and 200 mM ammonium sulfate, respectively.^44,45^ If we approximate the PEG in solution by a sphere with radius *R*, that can be calculated from obtained *R*_g_ by
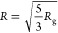
we get *R* in the range of 1.42–1.59 nm, and *D* = 2 × *R* is in the range of 2.84–3.18 nm. These observations are consistent with the dimensions estimated from *P*(*r*) and indicate that the chain observed in the 3D reconstruction might be PEG2000 interacting with the Hb molecule. While the resolution is too low to pinpoint the exact interaction site on Hb, we suppose that the interaction type aligns with those described earlier.

In the context of protein crystallization, PEG600–4000 are in the PEG size range that most often results in good-quality protein crystals.^46^ PEGs with *M*_w_ < 1000 are mainly used for the crystallization of membrane proteins, while PEGs with higher molecular weights show better results in the crystallization of soluble proteins^47^ (see the references in ref (47)). The work of Sato-Tomita and Shibayama demonstrated the variety of morphologies of Hb crystals obtained using PEGs as one of the components of crystallization mother liquors.^27^ When added to an aqueous solution of Hb, PEG induced “salting out” and crystallization of the protein under suitable conditions. Addition of PEG increased the overall solution viscosity, especially higher *M*_w_ PEGs.^27^ Based on our SAXS data, we have observed a decrease in the maximum size *D*_max_ of the Hb molecule as the size of the PEG increases. This phenomenon may be explained by the “dehydration effect” caused by higher-molecular-weight PEGs.^43^ Given sufficient PEG concentration (>15% w/v typically), Hb molecules begin to interact and assemble into ordered crystalline arrays. The crystallization is driven both by dehydration forces favoring the protein–protein interactions over water–protein ones as well as supersaturation effects. The effects of smaller PEGs are not similar to those of larger PEGs at the same content of PEG monomers in the sample, which may indicate slightly different mechanisms of precipitation by lower- and higher-molecular-weight PEGs.

### PEG and Radiation Protection

Lastly, we considered the impact of PEG on Hb stability during SAXS experiments. Synchrotron data revealed that PEG acts as a protective agent against Hb aggregation under X-ray exposure, with PEG600 demonstrating more potent stabilizing effects than PEG2000. Laboratory measurements, conducted with temperature stabilization at 20 °C, contrasted with the non-temperature-controlled environment at the synchrotron. Similar stabilizing effects of PEG were noted in BSA conformational stability studies, where PEG8000 binding led to thermal stabilization at optimal BSA/PEG molar ratios, as determined by DSC.^13^ The results indicate that PEGs can affect protein structure and stability, with the effects varying based on PEG size and concentration. Further investigation, perhaps using SANS, could provide more clarity on the PEG–protein interactions and help determine the best PEG types and concentrations for stabilizing specific proteins.

The differences observed in the scattering curves between Hb samples with and without PEGs can be attributed to several factors. PEG can alter the properties of the solvent, affecting the hydrogen bonding network and hydrophobic interactions within the Hb molecule. This can lead to changes in the protein’s tertiary and quaternary structures, which would be reflected in the SAXS data as changes in the overall shape and size of the protein. The crowding can promote a more compact protein structure that would alter the scattering intensity profile at different *q* values. Direct PEG-Hb surface interactions might induce and stabilize conformations distinct from those in PEG’s absence. The SAXS data support this as PEG presence prevents the low-*q* increase in scattering over time, typically a signature of radiation-induced aggregation. The molecular weight of PEG is a key factor of influence on Hb, with larger PEG molecules potentially inducing greater changes due to the increased possibility of interaction with the protein or more pronounced exclusion volume effects.

## Conclusions

We studied the effects of molecular weight and PEG concentration on the spatial structure of human hemoglobin at neutral pH using SAXS and SEC-SAXS. The commercial human Hb was characterized by SAXS coupled with SEC. The results demonstrated that at neutral pH, Hb has a tetrameric structure, and the dummy atom model derived from experimental data is in good agreement with the PDB crystallographic structure of *met*Hb. Short-chain PEG600 up to 10% (w/v) concentration exhibits a stabilizing effect on Hb compactness without substantially changing the spatial structure of the protein. 5% (w/v) PEG with molecular weights of 2000 and 4000 slightly decreases *D*_max_ with an almost unchanged *R*_g_ compared to pure Hb. This could indicate a decrease in the water shell caused by the ability of longer PEGs to dehydrate protein molecules. A 10% (w/v) content of PEG2000 in the sample leads to the interaction of the PEG molecule with Hb with the formation of a complex without distortion of the spatial structure of the protein. The data obtained allow a deeper understanding of the effect of PEG on the protein structural organization and complement the existing information on the interaction of PEGs with proteins. Future experiments using techniques like isothermal titration calorimetry or DSC could provide more direct evidence of changes in protein hydration. Combining these experimental results with MD simulations could help elucidate the mechanism underlying the stability or destabilization of hemoglobin.

## Data Availability

The experimental data were uploaded to the Zenodo (raw data) (10.5281/zenodo.11395807) and SASBDB (reduced data).

## Supporting Information Available

All experimental parameters and data analysis results were uploaded to the Zenodo (10.5281/zenodo.11395807) and SASBDB (PDF)
