# Supplementary material for: Hemoglobin–PEG Interactions Probed by Small-Angle X-ray Scattering: Insights for Crystallization and Diagnostics Applications
Source: J Phys Chem B. 2024 Sep 10;128(38):9262–73. doi: 10.1021/acs.jpcb.4c03003 (PMC11440596; doi:10.1021/acs.jpcb.4c03003)
Supplement: Supplementary file 1 — jp4c03003_si_001.pdf [file jp4c03003_si_001.pdf]

Supporting Information

Hemoglobin-PEG Interactions Probed by Small-Angle X-ray Scattering: Insights for Crystallization and Diagnostics Applications

Iuliia Baranova[1,2], Angelina Angelova[3], Jan Stransky[4], Jakob Andreasson[1], Borislav Angelov[1]*

[1]Extreme Light Infrastructure ERIC, Za Radnicí 835, Dolní Břežany, 252 41, Czech Republic,

[2]Charles University, Faculty of Mathematics and Physics, Ke Karlovu 3, Prague, 121 16, Czech Republic,

[3]Université Paris-Saclay, CNRS, Institut Galien Paris-Saclay, F-91400 Orsay, France,

[4]Institute of Biotechnology of the Czech Academy of Sciences, v.v.i., Prumyslová 595, Vestec, 252 50, Czech Republic

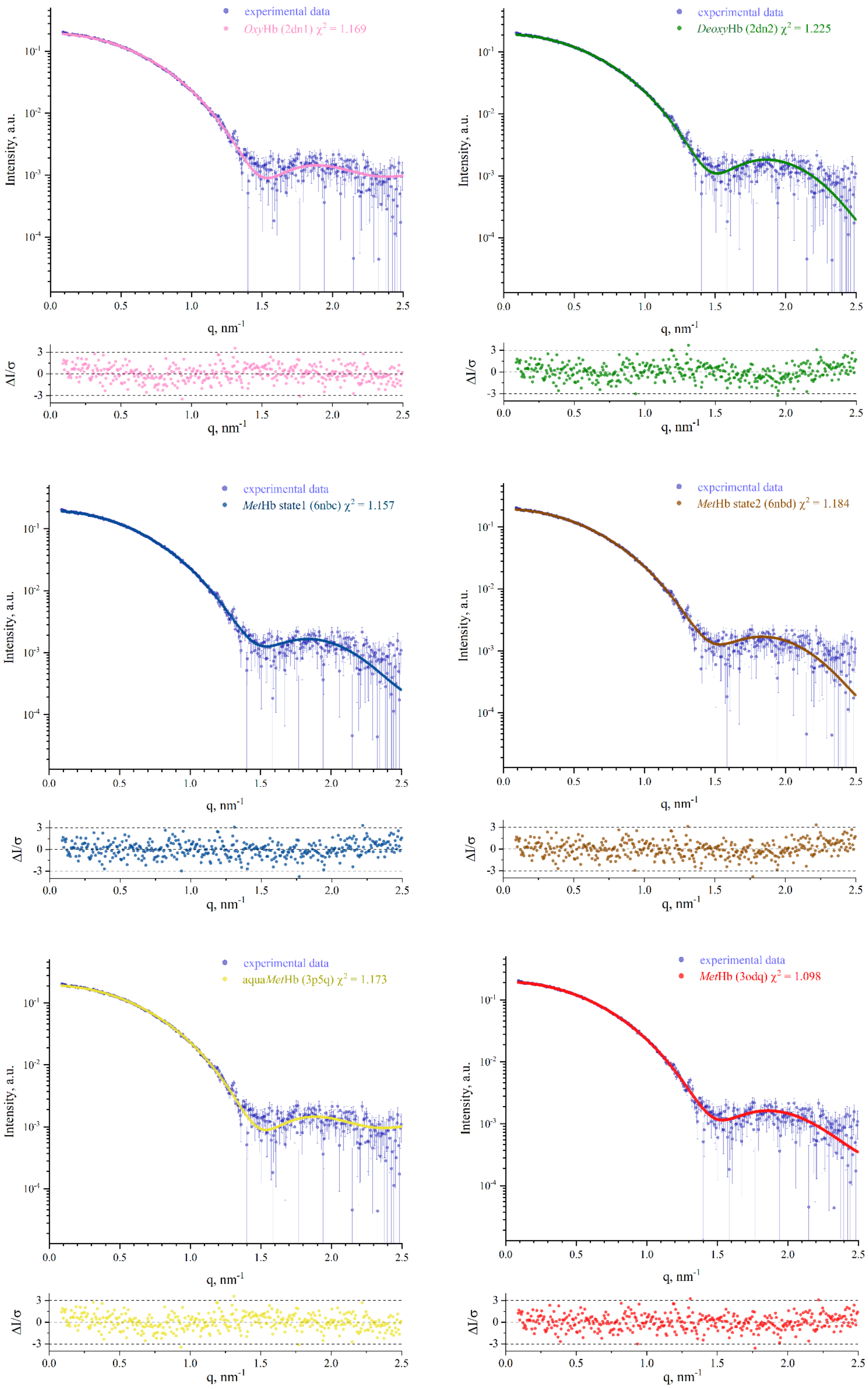
experimental data
OxyHb (2dn1) χ² = 1.169
experimental data
DeoxyHb (2dn2) χ² = 1.225
experimental data
MetHb state1 (6nbc) χ² = 1.157
experimental data
MetHb state2 (6nbd) χ² = 1.184
experimental data
aquaMetHb (3p5q) χ² = 1.173
experimental data
MetHb (3odq) χ² = 1.098
Intensity, a.u.
q, nm⁻¹
ΔI/σ

FIGURE

Figure S1. Averaged experimental SEC-SAXS data of the second elution peak (light purple circles) with theoretical scattering curves of various crystallographic structures from PDB calculated and fitted to experimental data using CRYSOL (*I(q)* in log scale *versus q*): *2dn1* – human *oxy*Hb (pink circles), *2dn2* – human *deoxy*Hb (green circles), *6nbc* – human *met*Hb state1 determined using single-particle cryo-EM (dark blue circles), *6nbd* – human *met*Hb state 2 deter-mined using single-particle cryo-EM (brown circles), *3p5q* – human R-state *aquomet*Hb (yellow circles), *3odq* – human *met*Hb indicative of fiber formation (red circles).

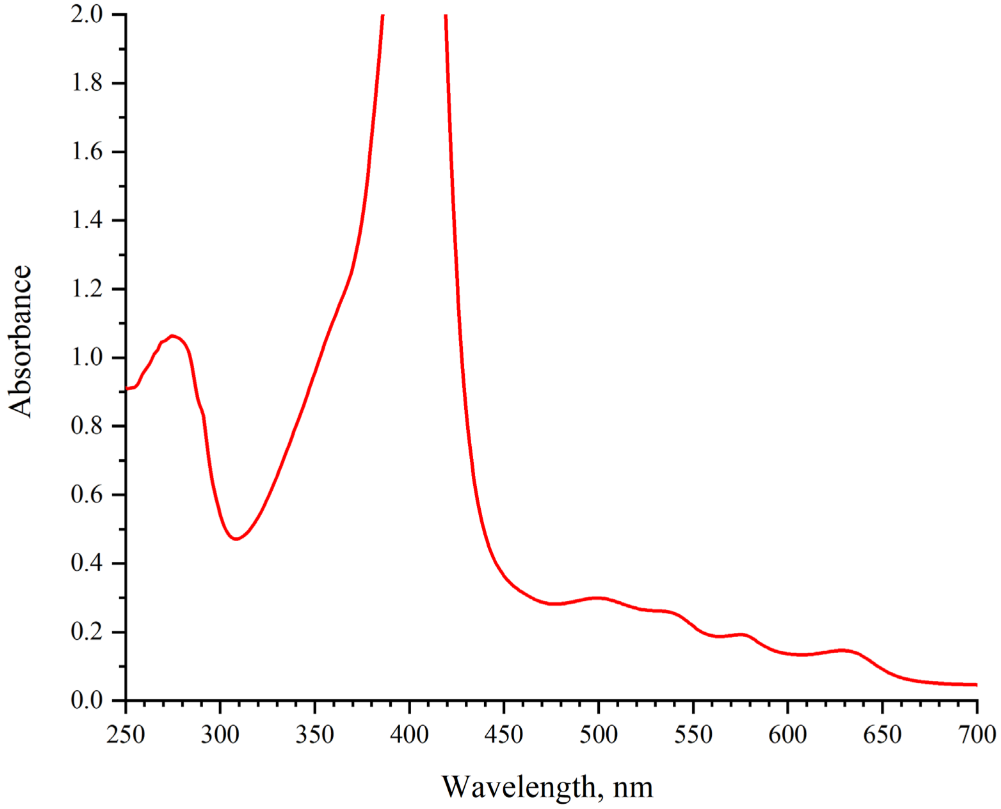

Figure S2. UV-Vis absorption spectrum of human haemoglobin (predominantly *Met* form).

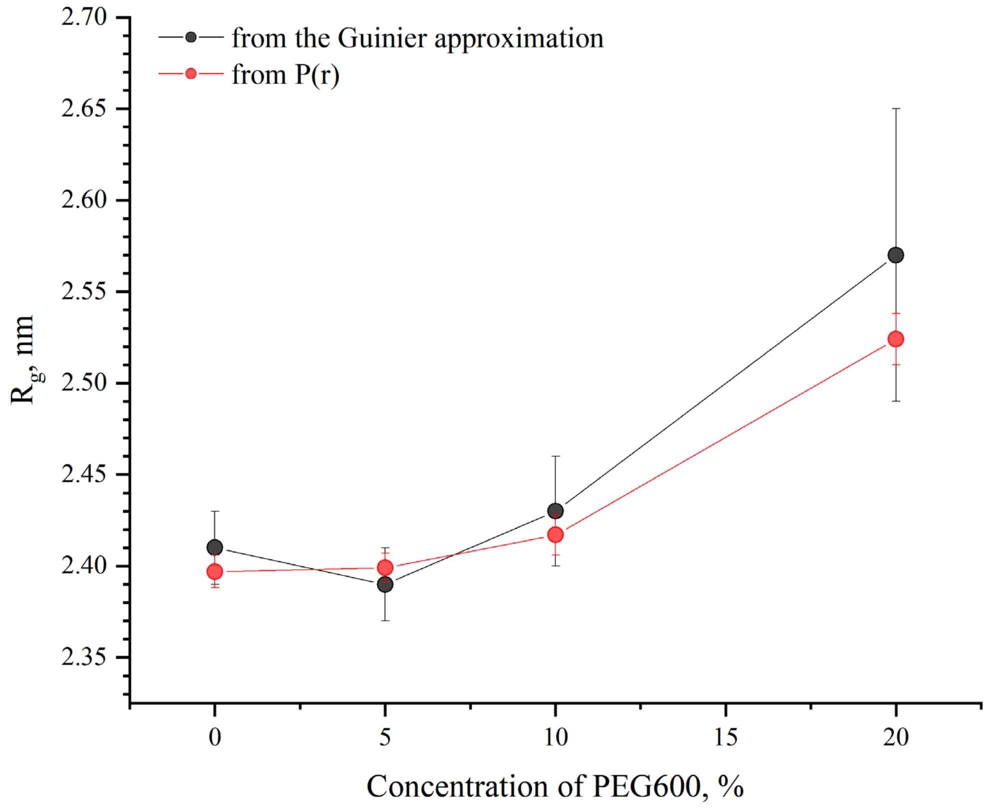

Figure S3. Dependence of R_g of Hb from concentration of PEG600: black circles represent R_g determined by Guinier analysis and red circles present R_g calculated from *P(r)*.

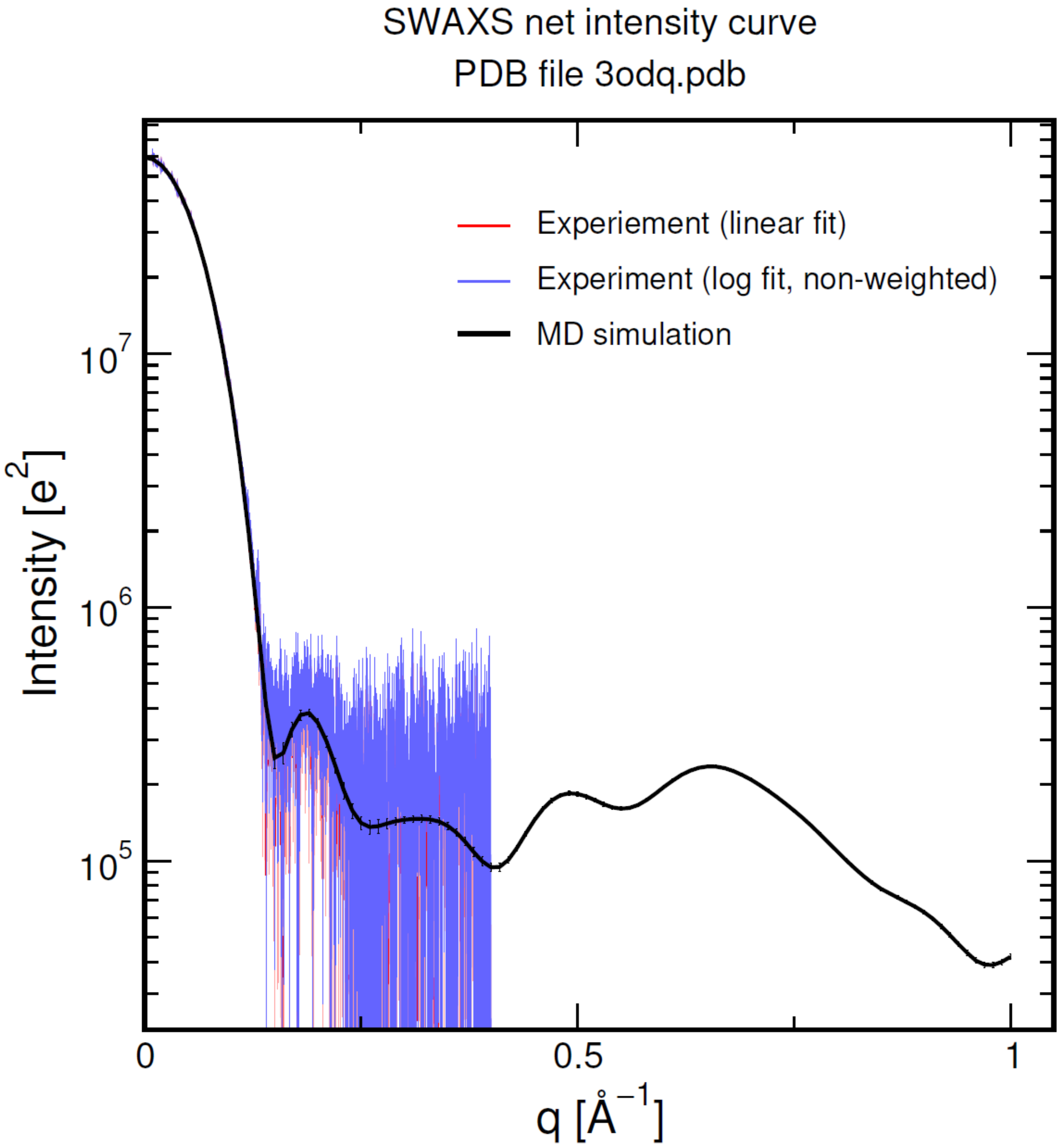

Figure S4. Fitting of the experimental solution SAXS data for hemoglobin with the WAXSiS generated SAXS/WAXS curve from molecular dynamics simulations (initial crystallographic structure PDB ID: *3odq*). Red and blue curves correspond to fitting on a linear and logarithmic scale respectively.

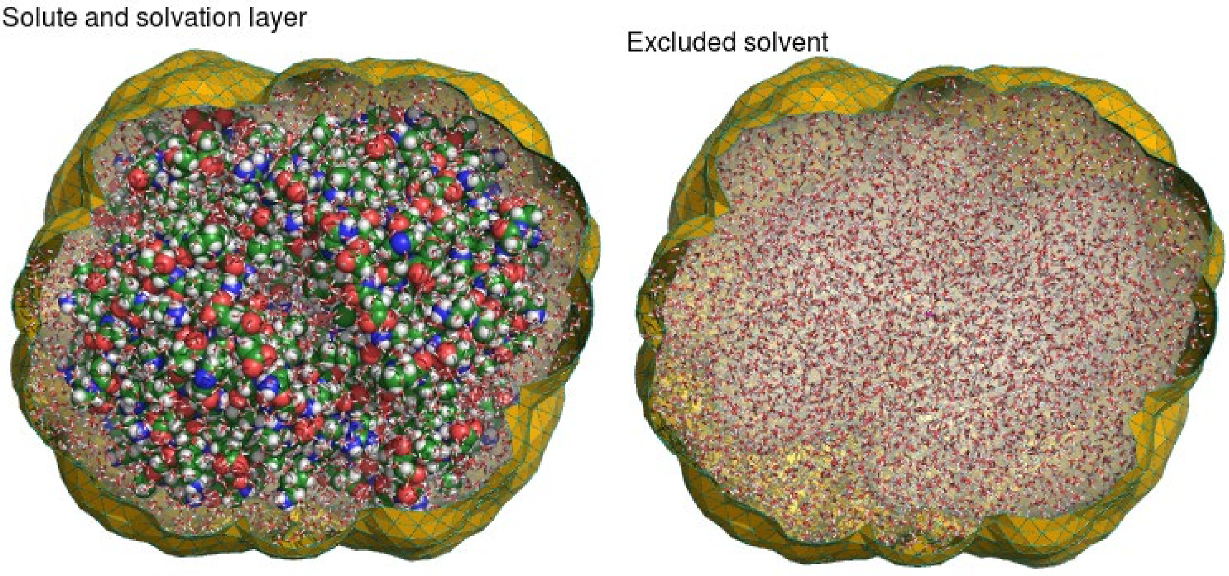

Figure S5. Visualization of the atomic model of hemoglobin in water solvent (left panel) and the excluded water solvent (right panel) used for MD simulations.

Table S1: Experimental parameters and data analysis results of SEC-SAXS and SAXS for Hb without and with PEGs

(1a) Sample details.

	Human Hemoglobin (Hb)
Organism	*Homo Sapiens*
Source (catalogue No. or reference)	Sigma-Aldrich (H7379)
UniProt sequence ID (residues in construct)	(P69905+P68871)*2
ῡ from chemical composition (cm³/g)	78205
Particle contrast from sequence and solvent constituents, Δρ ($\rho_{protein} - \rho_{solvent}$; 10^{10} cm^{-2})	1.836 (12.206-10.369)
M from chemical composition (Da)	64399*
SEC-SAXS column, 10×300 mm Superdex 75 Increase GL	
Loading concentration (mg/ml)	18**
Injection volume (µl)	500
Flow rate (ml/min)	0.05
Average C in combined data frames (mg/ml)	-
Solvent	100 mM Sodium Phosphate buffer, pH=7.0
Buffer - averaged frames	1-620
Sample - averaged frames	885-990

* M of the protein was calculated as the sum of M according to the a.a. sequence and M of 4 heme molecules.
** approximate weight estimate.

(1b) SAXS data-collection parameters.

Instrument/data processing	SAXSpoint 2.0 system from Anton Paar with Dectris Eiger 1M detector and Excillum MetalJet C2 X-ray source
Wavelength (Å)	1.34
Beam size (µm)	982.7×982.7
Camera length (mm)	792.6
q measurement range (nm^{-1})	0.0886-3.9971
Absolute scaling method	-
Normalization	Data were normalized to primary beam intensity measured using a semitransparent beamstop
Monitoring for radiation damage	-
Exposure time	Continious 15 s data-frame measurements of SEC elution
Sample configuration	SEC-SAXS with quartz capillary
Sample temperature (°C)	20

(1c) Software employed for SAXS data reduction, analysis and interpretation.

SAXS data reduction	*I(q) versus q* (custom house-developed code), solvent subtraction using *PRIMUS* (*ATSAS 3.2.0*)
Extinction coefficient estimate	*ProtParam* (via https://web.expasy.org/protparam/)
Calculation of Δρ and ῡ values	*MULCh* (via https://smb-research.smb.usyd.edu.au/NCVWeb/input.jsp)
Basic analyses: Guinier, *P(r)* , V_p	*PRIMUS, GNOM (ATSAS 3.2.0)*
Shape/bead modelling	*DAMMIF/N, DAMAVER (ATSAS 3.2.0)*
Atomic structure modelling	CRYSOL (ATSAS 3.3.0)
Missing sequence modelling	-
Three-dimensional graphic model representations	*ChimerX 1.6*

(1d) Structural parameters.

Guinier analysis	
I(0) (a.u.)	0.1964±0.0006
R_g (nm)	2.42±0.01
q_{min} (nm^{-1})	0.09439
qR_g min	0.27
qR_g max	1.29
Fidelity	0.27
M from *I(0)* (ratio to predicted)	-
P(r) analysis	
I(0) (a.u.)	0.1953±0.0004
R_g (nm)	2.381±0.005
d_{max} (nm)	6.8
q range (nm^{-1})	0.1170-2.5005
Total estimate from GNOM	0.94
M from *I(0)* (ratio to predicted value)	-
Porod volume ($Å^{-3}$) (ratio V_p /calculated M)	104861 (1.63)
V, M using the Fischer method (ratio of M to expected)	-

(1e) Shape model-fitting results.

DAMMIF (default parameters, 20 calculations)	
q range for fitting (nm^{-1})	0.0-2.501
Symmetry, anisotropy assumptions	P1, none
ICP (standart deviation)	2.54 (0.64)
χ^2 range	1.148-1.156
Constant adjustment to intensities	-
Resolution (from *SASRES*) (nm)	3.09±0.22
M estimate as 0.5 × volume of models (Da) (ratio to expected)	-
DAMMIN (default parameters)	
q range for fitting (nm^{-1})	0.0-2.5
Symmetry, anisotropy assumptions	P1, none
χ^2 , *CorMap P*-values	1.043, 0.56
Constant adjustment to intensities	-

(1f) Atomistic modelling.

Crystal structures	PDB 2dn1	PDB 2dn2	PDB 6nbc	PDB 6nbd	PDB 3p5q	PDB 3odq
q range for all modelling (nm^{-1})	0.0-2.5	0.0-2.5	0.0-2.5	0.0-2.5	0.0-2.5	0.0-2.5
CRYSOL						
Solvent density (e/Å^3)	0.361	0.361	0.361	0.361	0.361	0.361
Number of spherical harmonics	20	20	20	20	20	20
Constant subtraction allowed						
χ^2 , *CorMap P-values*	1.169, 0.012	1.255, 0.025	1.157, 0.095	1.184, 0.095	1.173, 0.012	1.098, 0.810
Predicted R_g (nm)	2.022	2.469	2.38	2.397	2.02	24.67
Vol (Å), R0 (Å), Dro (Å^{-3})	29279, 1.3, 0.1	58540, 0.8, 0.0	575064, 1.0, 0.1	517557, 0.8, 0.088	29320, 1.3, 0.1	58540, 0.8, 0.007

(2a) Sample details.

	Human Hemoglobin (Hb)
Organism	*Homo Sapiens*
Source (catalogue No. or reference)	Sigma-Aldrich (H7379)
UniProt sequence ID (residues in construct)	(P69905+P68871)*2
ῡ from chemical composition (cm³/g)	78205
Particle contrast from sequence and solvent constituents, Δρ ($\rho_{protein} - \rho_{solvent}$; 10^{10} cm^{-2})	-
M from chemical composition (Da)	64399*
Concentration of protein in the samples (mg/ml)	5**
Solvent and additives	100 mM Sodium Phosphate buffer, pH=7.0; PEG600, PEG2000, PEG4000 in concentrations of 5%, 10%, 20% (w/v)

* M of the protein was calculated as the sum of M according to the a.a. sequence and M of 4 heme molecules.

** the concentration of the stock was determined by a multi-component spectrophotometric method; the final concentration in the samples was achieved by dilution.

(2b) SAXS data-collection parameters.

Instrument/data processing	SAXSpoint 2.0 system from Anton Paar with Dectris Eiger 1M detector and Excillum MetalJet C2 X-ray source
Wavelength (Å)	1.34
Beam size (µm)	982.7×982.7
Camera length (mm)	825.6
q measurement range (nm^{-1})	0.0828-3.9971
Absolute scaling method	-
Normalization	Data were normalized to primary beam intensity measured using a semitransparent beamstop
Monitoring for radiation damage	-
Exposure time	30 sec × 30 frames per 1 sample
Sample configuration	1 mm quartz capillary
Sample temperature (°C)	20

(2c) Software employed for SAXS data reduction, analysis and interpretation.

SAXS data reduction	*I(q) versus q* (custom house-developed code), solvent subtraction using *PRIMUS* (*ATSAS 3.2.0*)
Extinction coefficient estimate	*ProtParam* (via https://web.expasy.org/protparam/)
Calculation of Δρ and ῡ values	*MULCh* (via https://smb-research.smb.usyd.edu.au/NCVWeb/input.jsp)
Basic analyses: Guinier, *P(r)* , V_p	*PRIMUS, GNOM (ATSAS 3.2.0)*
Shape/bead modelling	*DAMMIF (ATSAS 3.0.3) via BioXTAS RAW*
Atomic structure modelling	-
Missing sequence modelling	-
Three-dimensional graphic model representations	*ChimerX 1.6*

(2d) Structural parameters.

	Hb after 1 day	Hb with 5% (w/v) PEG600	Hb with 10% (w/v) PEG600	Hb with 20% (w/v) PEG600	Hb after 1 week	Hb with 5% (w/v)	Hb with 5% (w/v) PEG4000	Hb with 10% (w/v) PEG2000
Guinier analysis								
I(0) (a.u.)	0.1300±0.0009	0.1100±0.0007	0.0960±0.0008	0.0690±0.001	0.1200±0.0007	0.11±0.001	0.0970±0.0007	0.0820±0.0008
R_g (nm)	2.41±0.02	2.39±0.02	2.43±0.03	2.57±0.08	2.40±0.02	2.41±0.03	2.39±0.03	2.83±0.04
q_{min} (nm^{-1})	0.221	0.204	0.117	0.129	0.123	0.238	0.1462	0.13468
qR_g min	0.52	0.47	0.27	0.32	0.28	0.56	0.34	0.36
qR_g max	1.3	1.26	1.28	1.07	1.27	1.27	1.28	1.28
Fidelity	0.42	0.88	0.05	0.08	0.73	0.14	0.52	0.23
M from *I(0)* (ratio to predicted)	-	-	-	-	-	-	-	-
P(r) analysis								
I(0) (a.u.)	0.1260±0.0006	0.1103±0.0005	0.0960±0.0005	0.0671±0.0004	0.1205±0.0004	0.1083±0.0004	0.0960±0.0004	0.0828±0.0008
R_g (nm)	2.397±0.009	2.399±0.008	2.417±0.011	2.524±0.014	2.416±0.007	2.358±0.007	2.318±0.008	2.912±0.043
d_{max} (nm)	6.65	6.71	7.18	7.6	6.8	6.5	6.12	11.3
q range (nm^{-1})	0.2153-2.5063	0.2153-2.5063	0.1174-2.5063	0.1404-2.5063	0.1117-2.5063	0.1462-2.5063	0.1347-2.5063	0.1289-2.5063
Total estimate	0.8506	0.947	0.975	0.8983	0.9452	0.9773	0.9761	0.7791
M from *I(0)* (ratio to predicted value)	-	-	-	-	-	-	-	-
Porod volume ($Å^{-3}$) (ratio V_p/calculated M)	100185 (1.56)	105322 (1.64)	113570 (1.76)	113234 (1.76)	107469 (1.67)	91827.2 (1.43)	90543 (1.41)	133516 (2.01)*
V, M using the Fischer method (ratio of M to expected)	-	-	-	-	-	-	-	

* M of the particle was calculated as the sum of M of the protein molecule and M of the PEG2000 molecule.

(2e) Shape model-fitting results.

DAMMIF (default parameters, 20 calculations)	
q range for fitting (nm^{-1})	0.0-2.51
Symmetry, anisotropy assumptions	P1, none
NSD (standart deviation)	0.777 (0.011)
χ^2 range	1.071-1.08
Constant adjustment to intensities	-
Resolution (from *SASRES*) (nm)	3.2±0.2
M estimate as 0.5 × volume of models (Da) (ratio to expected)	51000 (0.77)
